# Mesenchymal Stromal Cells Improve Salivary Function and Reduce Lymphocytic Infiltrates in Mice with Sjögren's-Like Disease

**DOI:** 10.1371/journal.pone.0038615

**Published:** 2012-06-07

**Authors:** Saeed Khalili, Younan Liu, Mara Kornete, Nienke Roescher, Shohta Kodama, Alan Peterson, Ciriaco A. Piccirillo, Simon D. Tran

**Affiliations:** 1 Faculty of Dentistry, McGill University, Montreal, Quebec, Canada; 2 Department of Microbiology and Immunology, and FOCIS Centre of Excellence, Centre, Montreal, Quebec, Canada; 3 Academic Medical Centre, University of Amsterdam, Amsterdam, The Netherlands; 4 Department of Regenerative Medicine and Transplantation, Faculty of Medicine, Fukuoka University, Fukuoka, Japan; 5 Molecular Oncology, Faculty of Medicine, McGill University, Montreal, Quebec, Canada; National Institute of Dental and Craniofacial Research, United States of America

## Abstract

**Background:**

Non-obese diabetic (NOD) mice develop Sjögren's-like disease (SS-like) with loss of saliva flow and increased lymphocytic infiltrates in salivary glands (SGs). There are recent reports using multipotent mesenchymal stromal cells (MSCs) as a therapeutic strategy for autoimmune diseases due to their anti-inflammatory and immunomodulatory capabilities. This paper proposed a combined immuno- and cell-based therapy consisting of: A) an injection of complete Freund's adjuvant (CFA) to eradicate autoreactive T lymphocytes, and B) transplantations of MSCs to reselect lymphocytes. The objective of this was to test the effectiveness of CD45^−^/TER119^−^ cells (MSCs) in re-establishing salivary function and in reducing the number of lymphocytic infiltrates (foci) in SGs. The second objective was to study if the mechanisms underlying a decrease in inflammation (focus score) was due to CFA, MSCs, or CFA+MSCs combined.

**Methodology/Principal Findings:**

Donor MSCs were isolated from bones of male transgenic eGFP mice. Eight week-old female NOD mice received one of the following treatments: insulin, CFA, MSC, or CFA+MSC (combined therapy). Mice were followed for 14 weeks post-therapy. CD45^−^/TER119^−^ cells demonstrated characteristics of MSCs as they were positive for Sca-1, CD106, CD105, CD73, CD29, CD44, negative for CD45, TER119, CD11b, had high number of CFU-F, and differentiated into osteocytes, chondrocytes and adipocytes. Both MSC and MSC+CFA groups prevented loss of saliva flow and reduced lymphocytic infiltrations in SGs. Moreover, the influx of T and B cells decreased in all foci in MSC and MSC+CFA groups, while the frequency of Foxp3^+^ (T_reg_) cell was increased. MSC-therapy alone reduced inflammation (TNF-α, TGF-β), but the combination of MSC+CFA reduced inflammation and increased the regenerative potential of SGs (FGF-2, EGF).

**Conclusions/Significance:**

The combined use of MSC+CFA was effective in both preventing saliva secretion loss and reducing lymphocytic influx in salivary glands.

## Introduction

Sjögren's syndrome (SS) is a chronic autoimmune disease characterized by infiltrates of lymphocytes in the salivary glands [1], [2]. In SS the immune system attacks the salivary glands, particularly the acinar cells. This leads to a loss of saliva secretion and consequently patients' quality of life is severely compromised due to xerostomia (dry mouth), dental caries, and oral infections [2], [3], [4], [5]. Unfortunately, there is no suitable treatment for SS. Current pharmacological therapy that depends on the stimulation of residual acinar cells frequently fails, since in many cases, all the salivary secretory tissue has already been lost [6]. Regeneration of destroyed salivary glands or restoration of their function would greatly improve the quality of life for these patients.

The non-obese diabetic (NOD) mouse is a frequently used animal model to study Sjögren's-like disease (SS-like) as it exhibits infiltrates of lymphocytes in the salivary glands (sialadenitis) with a gradual loss of salivary function [1], [7], [8], [9]. The reduced saliva output is similar to what is observed in SS patients [8]. Our group recently reported a two-step combined immuno- and cell-based therapy that restored saliva flow in NOD mice with SS-like disease [7]. The first step (immune-modifying therapy) consisted of one injection of complete Freund's adjuvant (CFA) to increase the levels of endogenous tumor necrosis factor alpha (TNFα) to eradicate autoreactive T lymphocytes through apoptosis. The second step (cell therapy) was injections/transplantation of major histocompatibility complex (MHC) class I-matched bone marrow cells from healthy mice to reselect lymphocytes [8], [10], [11]. Although saliva secretion improved in NOD mice treated by our combined immuno- and cell-based therapy, no differences were observed in focus score (number of lymphocytic infiltrates) [7]. We concluded that CFA was insufficient to decrease the inflammatory cell infiltrates in SGs and started to investigate for additional approaches to our proposed combined therapy. Recent evidence from our collaborators (DL Faustman and S Kodama) indicated that multipotent stem cells of non-lymphoid lineage (CD45-negative; CD45**^−^**) from the spleen contributed to the regeneration of bone, inner ear, cranial nerves, islets, hearts, and of particular interest to our work, salivary glands [12], [13], [14], [15]. The spleen and bone marrow are closely related organs, and both are among the first sites of hematopoiesis during gestation. However spleen cells are not easily obtained from patients, except from trauma cases. Bone marrow cells are clinically easier to harvest, such as from needle aspirates, and can be expanded in large numbers. CD45**^−^** cells in bone marrow include the population of multipotent mesenchymal stromal cells (MSCs). There are recent reports on the use of MSCs, due to their anti-inflammatory and immunomodulatory capabilities, as a therapeutic strategy for autoimmune diseases, such as rheumatoid arthritis, multiple sclerosis, type I diabetes, and systemic lupus erythematosus (SLE) [16], [17], [18]. Our initial study [7] used unfractionated bone marrow cells (i.e. containing a mixture of both hematopoietic and mesenchymal cells) and we could not conclude if the observed re-establishment of saliva flow (therapeutic clinical effect) was due to cells from the mesenchymal lineage or from the hematopoietic one. Therefore the primary objective of this study was to assess the effectiveness of an enriched fraction of CD45^−^/TER119^−^ cells (MSCs) in re-establishing salivary function and in reducing the number of lymphocytic infiltrates (foci) in salivary glands. CD45 is a trans-membrane protein tyrosine phosphatase. It is expressed at high levels on the cell surface of all nucleated hematopoietic cells and their precursors [19]. The TER119 antigen is associated with Glycophorin A on erythrocytes and is used as a marker for erythroid cells from the early pro-erythroblast to mature erythrocyte stages of development [20]. Non-hematopoietic lineage is confirmed by the absence of CD45 (CD45^−^) and TER119 (TER119^−^) [21]. Our second objective was to study if the mechanism underlying a decrease in inflammation (focus score) was due to CFA, MSCs, or a combination of CFA+MSCs. We hypothesized that CD45^−^/TER119^−^ cells could be used to develop therapies for SS patients.

## Results

### 1- Both combined and MSCs therapies preserved normal salivary secretion

**Figure 1:**
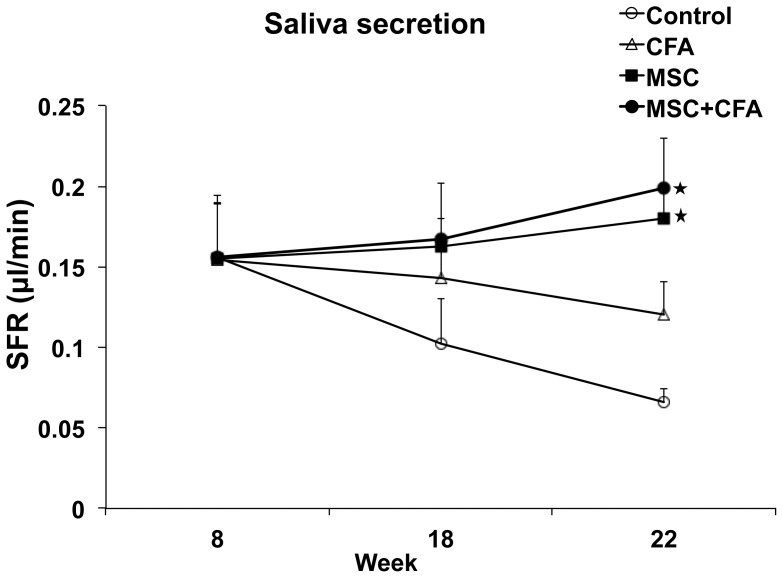
Salivary flow rates (SFRs) of NOD mice. SFRs in MSC+CFA (black circle; n = 10) and MSC (black square; n = 5) groups did not decrease during the follow-up period (22 wk of age) and were significantly higher than SFRs of CFA-treated or control NOD groups (n = 5 per group; *P*<0.05). SFRs in CFA (triangular) or control (untreated; open circle) groups continued to decrease during the follow-up period (* *P*<0.05).

Salivary gland function was assessed by measuring the salivary flow rate (SFR) of 8-week old NOD mice at baseline and for 14 weeks post-therapy. SFR directly reflects function of the glands and its decrease is the major clinical finding in patients with SS. In untreated NOD mice, SFR continued to deteriorate during the 14 weeks follow-up period (Figure 1). SFR of CFA-treated NOD mice (with no cell transplants) also deteriorated but to a lesser extent. SFRs of NOD mice that received the combined immuno- and cell-based therapy (CD45^−^/TER119^−^ MSCs with CFA) or the cell-based therapy alone (CD45^−^/TER119^−^ MSCs) were maintained during the 14 weeks post-therapy period and were statistically higher than SFRs of untreated and CFA-treated NOD mice (*P*<0.05; Figure 1). The composition and quality of saliva was examined for EGF, amylase, and total protein concentrations. These levels did not change significantly between pre-therapy (8-week NOD mice, prior to SS-like) and post-therapy (Figure S1). Blood glucose levels were monitored as NOD mice are known to develop type I diabetes. Sixty percent of control NOD mice (only receiving injections of insulin) developed diabetes at 22 weeks of age (Figure S2; P<0.05). However, 80%–90% NOD mice in the 3 other treated groups (CFA, MSC, and MSC+CFA) showed a relatively stable blood sugar level (normoglycemia) until 22 weeks of age.

### 2- MSC therapy decreased lymphocyte infiltration and foci formation in salivary gland

**Figure 2:**
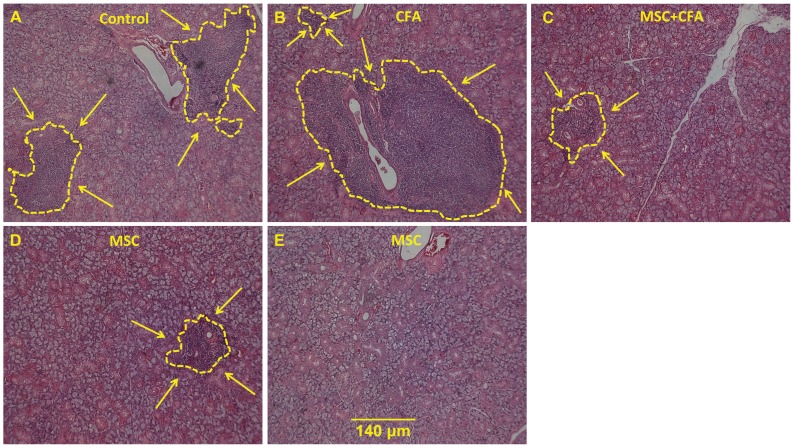
Infiltrate of lymphocytes in salivary glands of NOD mice. **A–E:** H&E staining showing size of the lymphocytic infiltrations (delineated with a yellow line and arrows) in NOD that were untreated (A), CFA-treated (B), MSC+CFA (C), MSC cells (D). In 2 of the 5 NOD mice transplanted with MSC only, no lymphocytic infiltrates were noted (E). Scale bar: 140 um for all images. **F:** Graph showing distribution of focus score (n = 5 to 9 mice per group). The mean focus score was significantly higher in control (3.24) versus CFA (1.81), MSC (1.23), and MSC+CFA (1.8) NOD groups (* *P* = 0.02).

To explore the mechanism underlying the preservation of SFR in NOD mice, salivary tissues were histologically analyzed for inflammatory signs, gene expression levels and cell chimerism. The focus scores (number of lymphocytic infiltrates in salivary tissue; Figure 2) of the CFA (1.81), MSC+CFA (1.8), and MSC (1.23) groups were all lower than the focus score of control NOD mice (3.24; *P*<0.05). The size of the lymphocytic infiltrate was smaller in NOD mice that received MSCs with or without CFA, when compared to untreated or CFA-treated mice (Figure 2 A, B, C, D, E). Interestingly, no lymphocytic infiltrates were detected in two out of five NOD mice transplanted with MSCs only (Figure 2E).

### 3- MSC therapy lessened inflammation while combined MSC+CFA therapy favored salivary tissue regeneration

**Figure 3:**
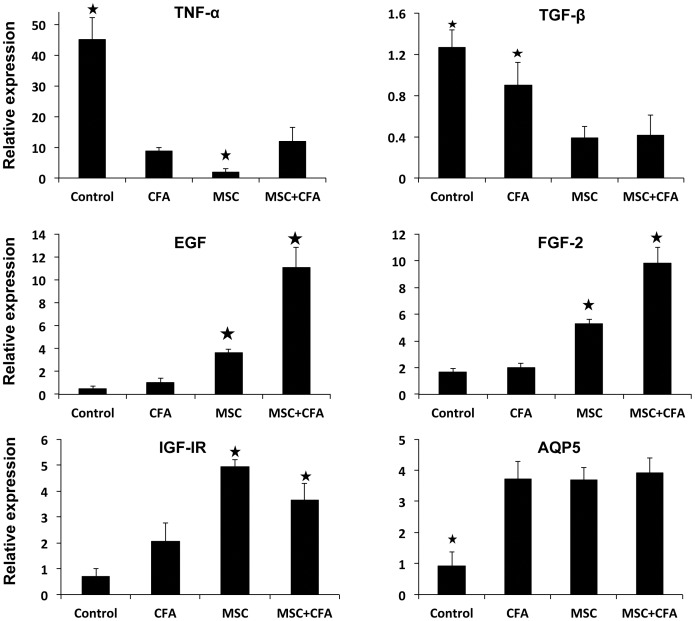
Relative gene expression of key inflammatory cytokines and growth factors involved in the pathogenesis of Sjögren's syndrome. TNF-α and TGF-β mRNAs were downregulated in CFA-treated and MSC+CFA versus control NOD mice (upper panel; * *P*<0.05). NOD mice transplanted with only MSCs had the lowest expression of TNF-α (* *P*<0.05). EGF, IGF-IR and FGF-2 mRNAs (middle and lower panels) were upregulated (* *P*<0.05) in MSC and MSC+CFA groups. The combined therapy (MSC+CFA) had the highest upregulation for regenerative genes (EGF, FGF-2). n = 4 to 8 mice per group.

**Figure 4:**
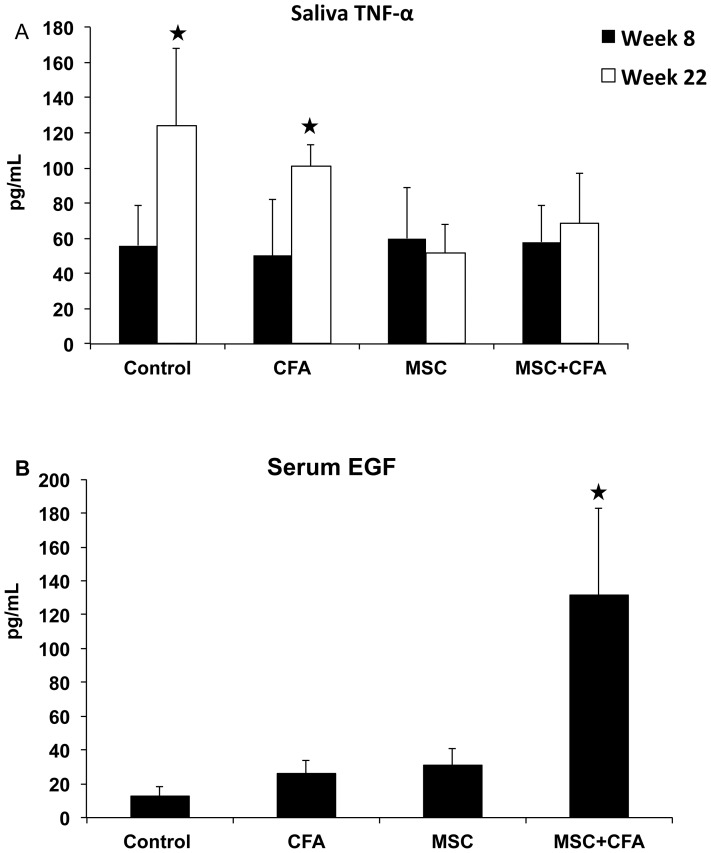
Measurements of TNFα and EGF concentrations in saliva and serum. *Upper panel (A)*, as expected, the level of TNFα in saliva was significantly higher (*P<0.05) in non-treated NOD mice at week 22 (established Sjögren's-like disease) when compared to week 8 (before disease onset). At the end of the experimentation period (week 22), treated MSC and MSC+CFA mice had two times less TNFα levels than non-treated NOD (*P<0.05). *Lower panel (B)*, concentrations of serum EGF was significantly higher in MSC+CFA group versus control, CFA, and MSC groups (n = 4 to 8 mice per group; *P*<0.05).

Expression of several important growth factor/cytokine genes involved in the pathogenesis of SS (TNF-α, TGF-β1) and in salivary tissue regeneration/homeostasis (EGF, FGF2, IGF-IR, AQP5) was measured (Figure 3). TNF-α mRNA were ∼4 fold lower in CFA-treated and MSC+CFA versus control NOD mice (Figure 3 upper panel; *P*<0.05). NOD mice transplanted with only MSCs had the lowest expression of TNF-α (10 fold lower than control NOD and 5 fold lower than CFA or MSC+CFA; *P*<0.05). TNF-α concentration in saliva of MSC and MSC+CFA groups were twice lower than those of NOD controls (Figure 4 upper panel; *P*<0.05). Transforming growth factor β1 (TGF-β1) mRNA accumulated 2.5 fold lower in MSC and MSC+CFA mice versus control NOD (Figure 3; *P*<0.05). Epidermal growth factor (EGF) and fibroblast growth factor-2 (FGF-2) were 3 to 4 fold upregulated in MSC group when compared to controls (Figure 3; *P*<0.05). NOD treated with the combined immuno- and cell-based therapy (MSC+CFA) showed the highest upregulation of EGF and FGF2 (10 and 5-fold, respectively). Insulin-like growth factor receptor I (IGF-IR) was upregulated (4–5 fold) in the MSC and MSC+CFA groups when compared to control NOD (*P*<0.05). The water channel protein, aquaporin-5 (AQP5), was ∼4 fold upregulated in treated animals (CFA alone, MSC with or without CFA) (*P*<0.05). The concentration of serum EGF was 10 times higher in the MSC+CFA treated group when compared to control NOD, and 5 times higher than the two other groups (only CFA, or only MSC) (Figure 4 lower panel; *P*<0.05).

### 4- MSC and combined therapy decreased the composition of T and B lymphocytes in foci, but increased the frequency of Foxp3^+^ T_reg_ cells

**Figure 5:**
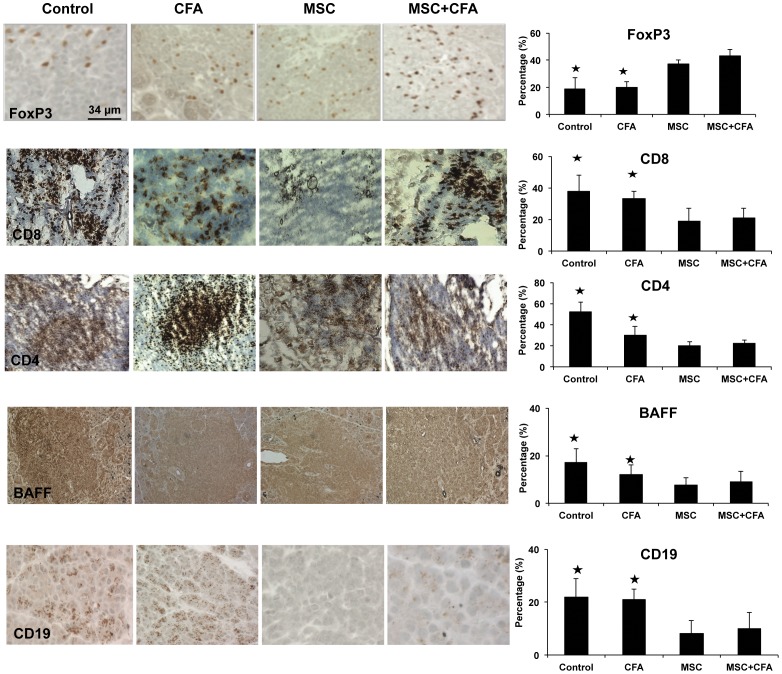
Immunohistochemical staining of salivary glands at week 22 (n = 3 to 5 mice per group). Cells positive for Foxp3^+^ (regulatory T cells), CD8^+^ (T cytotoxic), CD4^+^ (T helper), BAFF and CD19^+^ (B cells) appeared brown in the foci. Graphs on the right represent percentage of positive cells occupying the area of the focus score. Regulatory T cells (Foxp3^+^) increased in MSC and MSC+CFA groups (*P*<0.05) while T cytotoxic (CD8^+^), T helper (CD4^+^), BAFF, and B cell (CD19^+^) decreased after MSC or MSC+CFA treatment, when compared to control and CFA groups (* *P*<0.05).

Proportions of immune cells in focus score, such as T helper (CD4^+^), T cytotoxic (CD8^+^), B cell (CD19^+^), and BAFF, was fewer in MSC and MSC+CFA groups when compared to control or CFA groups (Figure 5; *P*<0.05). However, regulatory T cells (T_reg_ Foxp3^+^) cells increased in MSC and MSC+CFA groups (*P*<0.05). No significant difference was found for natural killer (CD49b^+^) and plasmacytoid dendritic cells (CD11c^+^).

### 5- Transplanted CD45^−^/TER119^−^ cells have characteristics of multipotent mesenchymal stromal cells (MSCs)

**Figure 6:**
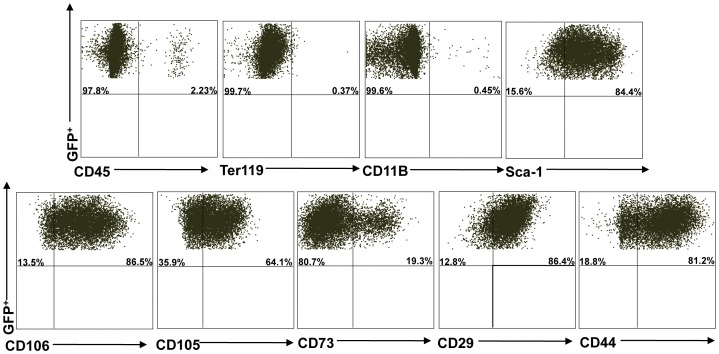
Flow cytometry analysis of CD45^−^/Ter119^−^ cells. After 3 passages, CD45^−^/Ter119^−^ cells were stained with the following surface markers: CD45, Ter119, CD11B, Sca-1, CD106, CD105, CD73, CD29 and CD44. Data are representative of at least three separate experiments. This experiment shows 97.8% CD45^−^, 99.7% TER119^−^, 99.6% CD11b^−^ and 84.4% Sca1^+^, 86.5% CD106^+^, 64.1% CD105^+^, 19.3% CD73^+^, 86.4% CD29^+^, 81.2% CD44^+^ cells.

**Figure 7:**
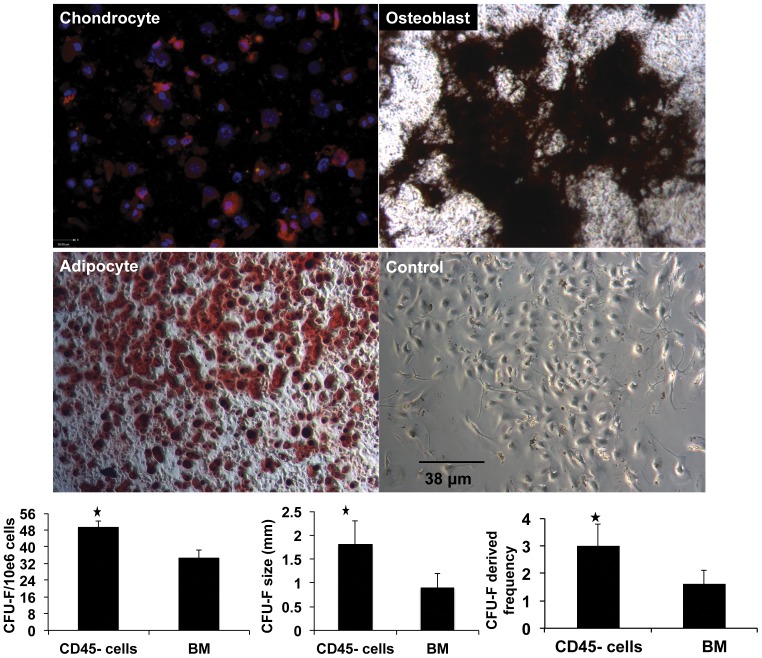
Functional assays for multipotent mesenchymal stromal cell (MSCs). Photomicrographs of CD45^−^/TER119^−^ cells that undergone chondrocytic, osteogenic and adipocytic differentiation when cultured in differentiation media. Lower right photomicrograph represents CD45^−^/TER119^−^ cells cultured in non-differentiating media (negative control). Collagen II (red) and DAPI (blue nuclei) are used to identify chondrocytes. Von kossa (black) and oil red stainings were used to identify osteoblasts and adipocytes, respectively. Graphs represent the number, size, and frequency of colony-forming unit fibroblasts (CFU-F) of CD45^−^/TER119^−^ cells versus whole bone marrow cells. All three graphs show that CD45^−^/TER119^−^ cells had higher CFU-F than whole bone marrow cells (* *P*<0.05).

Multiparameter flow cytometry was used to characterize CD45^−^/TER119^−^ cells freshly isolated from the bones of eGFP mice (before cell culture; Figure S3), and then again after three passages in culture (before cell transplantation procedures; Figure 6). An antibody panel to identify mouse MSCs consisting of positive markers for Sca-1, CD106, CD105, CD73, CD29, CD44 and negative markers for CD45, TER119 and CD11b was used. Flow cytometry results (based on 3 experiments; mean± SD) of freshly isolated CD45^−^/TER119^−^ cells demonstrated following frequencies; 70.75%±11.52 CD45^−^, 98.45%±0.63 TER119^−^, 97.50%±0.56 CD11b^−^ and 46.85%±3.04 Sca1^+^, 62.75%±2.75 CD106^+^, 55.0%±3.67 CD105^+^, 47.7%±0.56 CD73^+^, 87.7%±2.82 CD29^+^, 40.9%±4.52 CD44^+^ (Figure S3). After three passages in culture, these adherent CD45^−^/TER119^−^ cells were enriched to 96.1%±5.09 CD45^−^, 97.93%±2.80 TER119^−^, 99.00%±0.84 CD11b^−^, and 83.45%±1.34 Sca1^+^, 73.72%±13.34 CD106^+^, 60.44%±9.91 CD105^+^, 23.92%±6.55 CD73^+^, 87.85%±2.05 CD29^+^, 84.45%±4.59 CD44^+^ (Figure 6). The number, size and frequency of colony-forming unit fibroblasts (CFU-F) were statistically higher in CD45^−^/TER119^−^ cells than unfractionated bone marrow cells (Figure 7; *P*<0.05). CD45^−^/TER119^−^ cells underwent osteogenic, chodrocytic and adipocytic differentiation when cultured in differentiating media (Figure 7). All these characteristics indicated that transplanted CD45^−^/TER119^−^ cells were multipotent mesenchymal stromal cells (MSCs).

### 6- Transplanted male-eGFP MSC donor cells did not engraft in salivary tissues of female NOD mice

**Figure 8:**
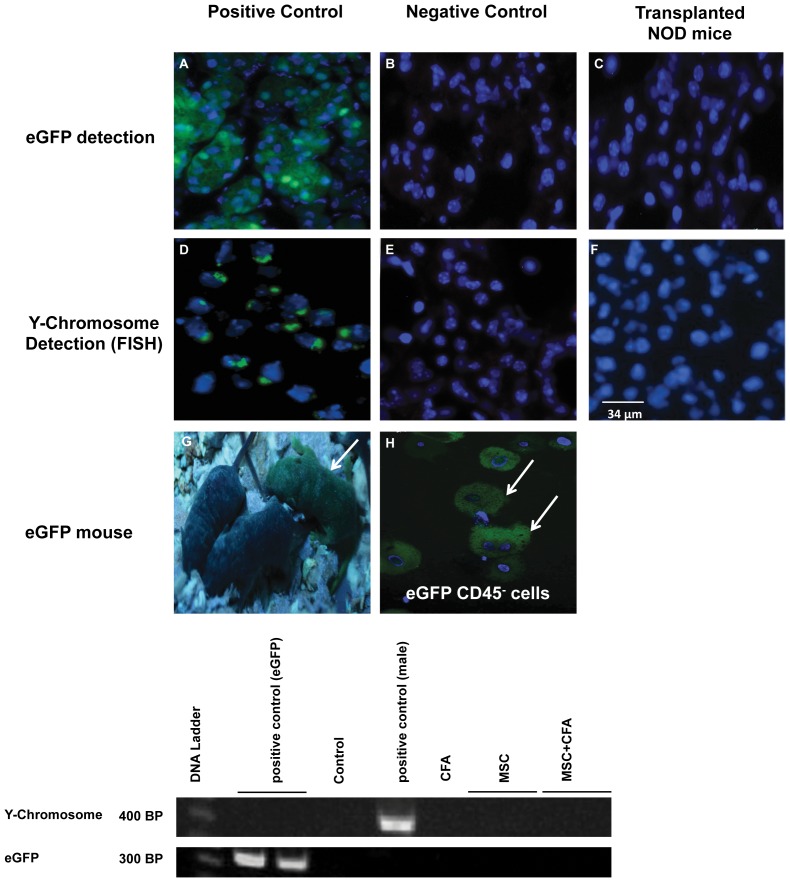
Absence of eGFP and male MSC (CD45^−^/TER119^−^) donor cells in salivary tissues of female NOD mice. Top panel: eGFP detection of MSC donor cells by immunostaining. (A) Salivary tissues from the eGFP donor mouse (positive control) versus (B) the non-eGFP mouse (negative control). (C) NOD mice transplanted with MSCs were negative for eGFP cells. Middle panel: Y-chromosome detection of male MSCs by FISH. (D) male and (E) female salivary tissues used as positive and negative controls, respectively. (F) Female NOD mice transplanted with male MSCs showed no Y-chromosome signal in their salivary glands. Bottom panel: (G) CByB6F1-eGFP transgenic mouse (arrow) and (H) the isolated donor eGFP MSCs before transplantation. Cell nuclei are stained in blue (Hoechst 33258). (I) PCR amplification did not detect the Y-chromosome and eGFP signal in salivary glands of MSCs transplanted NOD mice (n = 11).

Fluorescence in situ hybridization (FISH) and immunostaining were used to detect the male (Y-chromosome) and eGFP markers of transplanted MSCs in female NOD salivary tissues, respectively. No Y-chromosome or eGFP-positive cells were detected (Figure 8). Salivary tissues also were investigated by PCR for the presence of the Y-chromosome and eGFP; no signal was detected for either (Figure 8).

## Discussion

### The major findings of this study were as follows

1. Both MSC and MSC+CFA groups prevented loss of saliva flow and reduced lymphocytic infiltrations in salivary glands during the 14 weeks post-therapy period.
2. MSC therapy reduced inflammation (TNF-α, TGF-β, focus score).
3. Combined therapy (MSC+CFA) reduced inflammation and increased the regenerative potential of salivary glands (EGF, FGF2, IGF-IR).
4. Both MSC and combined therapy decreased the number of T and B cells in foci, while increasing regulatory T cells (T_reg_).
5. CD45^−^/TER119^−^ cells demonstrated characteristics of multipotent mesenchymal stromal cells (MSCs) as they were positive for markers Sca-1, CD106, CD105, CD73, CD29, CD44, negative for CD45, TER119, CD11b, had high number of CFU-F, and differentiated into osteocytes, chondrocytes and adipocytes.
6. A lack of male eGFP MSCs engrafted in the salivary parenchyma of transplanted female NOD mice suggested that tissue repair/regeneration originated from endogenous salivary cells.

Salivary secretion function was measured using salivary flow rate (SFR) pre- and post-cell transplantation. As expected, SFR of control NOD mice deteriorated during the follow-up period [22]. MSC-treated groups (with or without CFA) maintained their SFRs (Figure 1). SFR of CFA-treated mice also deteriorated, but to a lesser extent when compared to control NOD. There was no statistically significant difference in SFR between MSC-treated groups and the CFA group when NOD mice were at 18 weeks of age (Figure 1). We have observed, in this study and in our previous one [7], that CFA was helpful in restoring SFR for a relatively short-term but as the follow-up time was longer, this effect was lost. This may be due to the limited action/stimulus of CFA on salivary glands, as explained below.

Normally, autoreactive T cells are destroyed in the bone marrow or thymus during the process of negative selection. In autoimmune diseases, autoreactive T cells survive after having evaded negative selection. In the NOD mouse model, autoreactive T cells escape negative selection because of a proteasomal defect that alters protein processing, including processing of self-antigens for display on their cell surface. Without appropriate display of self-antigens, autoreactive T cells avoid being seen as ‘self’, and thus avoid targeted death. Their survival leads to onset or deteriorating of autoimmunity. But the underlying proteasomal defect in these cells also renders them exquisitely sensitive to TNFα-induced apoptosis. Because of the dysfunctional proteasome, autoreactive T cells of the activated type, do not form NF-κB. Lacking functional NF-κB in autoreactive T cells, they remain sensitive to TNFα-induced apoptosis. TNFα-induced apoptosis sensitivity can be exploited for therapeutic purposes. Several studies in the NOD mouse find that agents which induce TNF-α, such as bacillus Calmette-Guérin (BCG) or complete Freund's adjuvant (CFA, as in our study), have a similar effect to direct administration of TNF-α [10]. We and others have proposed a therapy using TNF-α (or TNFα-inducers such as CFA or BCG) to selectively kill autoreactive T cells in type I diabetes and SS [7], [23]. Treatment with TNF-α appears to offer a highly targeted strategy to destroy autoreactive activated T cells and interrupt the pathogenesis of autoimmunity [10], [24]. TNF-α does not appear to harm normal T cells or other tissues, presumably due to their active form of NF-κB. Our findings showed some (short-term) benefits of CFA for SS, but also that one single injection of CFA (to induce TNF-α) was insufficient to completely restore salivary function. TNF-α does not eliminate the autoreactive naïve T cells, which will become pathogenic on exposure to self-antigens. To prevent eventual self-tissue recognition and activation, autoreactive naïve T cells can be induced to die by an additional therapy such as by introducing MHC class I-matched cells (such as spleen, bone marrow, or MSCs) that could also be used to induce tissue repair/regeneration.

CD45^−^/TER119^−^ cells used in this study were tested for their stem cell properties. The “three minimum criteria" used to characterize MSCs are: a) plastic-adherent, b) specific surface antigen expression, and c) in vitro differentiation potential into osteocytes, chondrocytes and adipocytes [25]. Adherent CD45^−^/TER119^−^ cells were grown in MSC culture media for 3 passages. Despite the lack of a specific surface marker to identify MSC, the use of a panel of surface markers with flow cytometry is an accepted practice to characterize both primary and cultured MSCs. The positive markers for mouse MSCs are vascular cell adhesion molecule 1 (CD106), endoglin (CD105), 5′-nucleotidase (CD73), integrin beta-1 (CD29), hyaluronidase acid receptor (CD44), and stem cell antigen-1 (Sca-1) [26], [27]. These positive cell surface markers, together with the absence of hematopoietic markers such as Ter119, CD45, and CD11b can be used to characterize mouse MSCs [27]. CD45^−^/TER119^−^ cells used here were between 96–99% CD45, TER119 and CD11b negative (Figure 6). They were high (73–88%) for Sca1, CD106, CD29, CD44, moderate (60%) for CD105, and low (24%) for CD73. However, transplanted cells did not engraft or differentiate into salivary epithelial cells, as demonstrated by absence of male and eGFP cells in female NOD salivary glands (Figure 8). Recent studies reported functional improvements in several animal models and in certain patients occurred with transient appearances of the transplanted cells. This finding led to a new paradigm that transplanted cells delivered their therapeutic function, such as repairing tissue and influencing the immune response, through paracrine secretions and cell-cell interactions [16], [28]. MSCs were reported to secrete anti-inflammatory modulators and suppress the immune system [14], [15], [29], [30]. In autoimmune diseases, MSCs inhibited proliferation of T cytotoxic, natural killer and B cells, cytokine production, and differentiation of monocytes into antigen-presenting dendritic cells [17].

This study found that the proportion of immune cells in salivary glands was reduced in NOD treated with MSC (either with or without CFA; Figure 5). The numbers of T helper, T cytotoxic, and B cells were significantly decreased when compared to the CFA or control groups. The B-cell-activating factor (BAFF) promotes B-cell survival and allows autoreactive B cells to escape from apoptosis and to produce pathogenic autoantibodies [31]. A decrease in their numbers was observed in MSC and MSC+CFA treated groups. As T_reg_ cells expressed the critical fork head box p3 (Foxp3) transcription factor to maintain immune self-tolerance by actively suppressing self-reactive lymphocytes, we postulated that Foxp3^+^ T_reg_ cells inhibited the inflammation and disease [32], [33]. Consistently, recent studies suggest that treatment with MSCs augment Foxp3^+^ T_reg_ numbers in autoimmune rheumatic disease [34]. Interestingly, the frequency of Foxp3^+^ T_reg_ cells increased in MSC and MSC+CFA groups and correlated with the reduced inflammation observed in these groups.

Elevated production of inflammatory cytokines, such as TNF-α, is a common finding in the pathogenesis of SS [1], [2], [35], [36], [37], [38]. TGF-β is involved in salivary gland development and homeostasis; its production is increased in inflammatory and abnormal conditions [35]. MSC and MSC+CFA treated groups showed a down regulation of both TNF-α and TGF-β genes (Figure 3). The CFA group also showed a comparable down regulation of TNF-α as the MSC-treated groups and this may be due to its different mechanism of action (see explanation on CFA, above). Our results showed that the MSC-therapy alone was effective in reducing inflammation (TNF-α, TGF-β, focus score). However when it was combined with CFA, there was an added benefit for tissue repair/regeneration (FGF-2, EGF). FGF-2 is a modulator of cell proliferation, differentiation, angiogenesis, and wound healing [39]. FGF-2 increased re-epithelialization in salivary glands [40] and healing of oral mucosa ulcers [41]. EGF is a growth factor secreted by salivary ductal cells and is involved in the growth, regeneration, and maintenance of salivary glands. In addition, EGF has an anti-apoptotic function in salivary epithelial cells [42]. High levels of EGF were measured in the serum of MSC+CFA mice (Figure 4, lower panel). Salivary glands are known mainly as an exocrine gland. However, they also act (less well-known) as an endocrine gland and can secrete proteins/molecules into the blood stream [43]. The higher levels of serum EGF and its mRNA in MSC+CFA mice suggest that the preservation of salivary secretory function was a result of endogenous tissue regeneration/repair.

This paper focuses mainly on the therapeutic effects of MSC+CFA within the salivary glands of SS-like NOD mice. However since this is a systemic therapy likely to exert immunomodulatory functions, future studies investigating lymphocytes activation/differentiation in lymphoid tissues (such as cervical lymph nodes and the spleen) are desired to shed more light on the possible mechanisms of our combined therapy. Also measuring serum levels of autoantibodies and cytokines involved in Sjögren's syndrome would be useful.

## Materials and Methods

### Animals

All procedures with animals were carried out under protocols approved by the Facility Animal Care Committee of McGill University.

#### Recipients

8-week old female NOD mice with Sjögren's-like disease (SS-like) from Taconic Farms (Germantown, NY) were randomized into four groups and treated with: a) MSC plus CFA (MSC+CFA group; n = 10), b) MSC without CFA (MSC; n = 5), c) CFA injection only (CFA group; n = 5), or d) no cell injection, no CFA, but daily injections of insulin to control the blood sugar levels (Control untreated group; n = 5). Female NOD mice belonging to the CFA and MSC+CFA groups received one injection of CFA for TNF-α induction at baseline (8-week of age). NOD mice from MSC+CFA and MSC groups were transplanted with male eGFP MSCs bi-weekly for 2 weeks. The use of male eGFP donor cells allowed for the localization of Y-chromosome and eGFP cells in female NOD mice salivary tissue.

*Donors (CByB6F1-eGFP male) mice:* male GFP transgenic mouse [C57BL/6-TgH (ACTbEGFP)10sb/J] (stock number # 003291) were bred with female BALB/c mice (stock number # 00651) from Jackson Laboratory, NY. The transgenic mouse line has an “enhanced" GFP (eGFP) cDNA under the control of a chicken beta-actin promoter and cytomegalovirus enhancer makes all of the tissues, with the exception of erythrocytes and hair, appear green under excitation light (488 nm light source). To screen the newborns for the expression of eGFP, they were: A) illuminated with a UV light (UVP, UK cat no # 1199069) and B) genotyped by PCR. DNA was extracted (PureLink Genomic DNA mini kit, Invitrogen, USA, Cat no# K1820-02) from a tail biopsy and analyzed by PCR for the presence of the eGFP gene. The primers were (forward) 5′-GCA-CCA-TCT-TCT-TCA-AGG-ACG-AC-3′ and (reverse) 5′-CGT-GGT-AGA-AGA-AGT-TCC-TGC-TG-3′. PCR amplification was done as follows: 94°C for 7 minutes, 94°C for 1 minute, 59.6°C for 1 minute, and 72°C for 1.30 minute for 29 cycles; and 72°C for 10 minutes.

### Cells

#### Isolation and enrichment of CD45^−^/TER119^−^ cells from bone marrow and compact bone

eGFP transgenic donor mice were sacrificed. Muscles were removed to expose the femur and tibia. These bones were crushed according to the manufacturer's instruction (Cat No# 19771, Stem cells Technologies, Vancouver, BC). Unwanted hematopoietic (non-mesenchymal) cells were removed with antibodies directed against CD45 and TER119. These hematopoietic cells were bound to magnetic particles, separated using a magnet, and discarded. This strategy allowed for the negative selection of mesenchymal cells, which were CD45^−^/TER119^−^, from the compact bone and its marrow. The isolated cells were plated in 100 mm cell culture dish using MesenCult MSC basal medium and stimulatory supplements (Cat No#05511, Stem cells Technologies). These adherent cells were cultured for 3 passages (3 weeks).

#### Cell transplantation

1×10^7^ CD45^−^/TER119^−^ cells were injected into female NOD recipients through the tail vein, twice a week for two consecutive weeks. No cells were injected into NOD mice in the CFA or Control groups. CFA (Difco, Detroit, MI) was freshly mixed with an equal volume of physiological saline and injected (50 µl) into each hind footpad simultaneously with the first CD45^−^/TER119^−^ cells injection. CFA was also injected once in NOD mice of the CFA group. No CFA was injected in mice of the control group [7], [8].

#### Flow cytometry

Staining for surface phenotyping were done with the following fluorochrome-conjugated mAbs: anti-Ter119 (*TER-119*), anti-CD11b (*M1/70*), anti-CD106 (VCAM-1)(*429*), anti-CD105 (*MJ7/18*), anti- Ly-6A/E (Sca-1) (*D7*), anti-CD73 (*TY/11.8*), anti-CD29 (*eBioHMb1-1*), anti-CD44 (IM7) (eBioscience, San Diego, CA), anti-CD45 (*30-F11*), (BD Bioscience, Mississauga, Ontario). Data were acquired on a BD LSRFortessa (BD) and analyzed with Flowjo software (Tree Star). Flow cytometry experiments were repeated 3 times.

#### Functional assays for multipotent mesenchymal stromal cell (MSC)

The number, size, and frequency of Colony Forming Unit- Fibroblast (*CFU-F*) between cultured CD45^−^/TER119^−^ and bone marrow cells for 10–13 days were assessed using the CFU-F kit (Cat No # 28374, Stem cell technologies, BC. Canada). A colony forming unit is defined as >40 cells. CD45^−^/TER119^−^ cells were differentiated into osteocytes, adipocytes and chondrocytes (‘differentiation assay’) using stimulatory supplements (Cat No # 05503, Stem Cell Technologies, or # CCM006, R&D Systems, MN) according to the manufacturer's instructions. Von Kossa and oil red histochemical stainings were used to detect osteocytes and adipocytes, respectively. Immunofluorescence staining to collagen II was used to identify chondrocytes (Collagen II antibody, # AF3615; Northern Lights 557-conjugated anti-sheep IgG, # NL010, R&D Systems, MN). All experiments were done in duplicate and repeated 3 times.

### Measurements of salivary function

#### Secretory function of the salivary glands (salivary flow rate; SFR)

SFR was obtained by inducing mild gas anesthesia to NOD mice with 5% Isoflurane, 5% Halothane, and 0.5–1 L/min oxygen (as per animal facility protocols at McGill University). Whole saliva was collected after stimulation of secretion using 0.5 mg pilocarpine/kg body weight administered subcutaneously. Saliva was obtained from the oral cavity by micropipette, placed into pre-weighed 0.5-ml microcentrifuge tubes. Saliva was collected for a 10-minute period and its volume determined gravimetrically. SFR was determined at week 8 (baseline), week 18, and week 22 of age [7].

#### Analysis of saliva quality/composition

The concentration of proteins in saliva was measured by the bicinchoninic acid assay method (BCA; Thermo Fisher Scientific Cat no # 23225). Levels of epidermal growth factor (EGF) were measured by ELISA (R&D Systems, Minneapolis, MN, Cat no # MGE00). Amylase activity was measured with a colorimetric method (Salimetrics, State College, PA, Cat no # 1-1902). All measurements were performed at the beginning (baseline; week 8) and at the end of the study (week 22 of age).

### Salivary tissue and serum analysis

1- **Fluorescence in situ hybridization (FISH).** Was done, as previously described [7], to detect male transplanted cells in female NOD mice.

2- **PCR**. was also used to detect male transplanted cells in female NOD mice. DNA was extracted from submandibular glands and purified (PureLink Genomic DNA Mini kit, cat K1820-02, Invitrogen). The sequences for the Y-chromosome sense primer were 5′ AAT-TGA-CAG-CAT-CTA-CGT-ACT-GGA-GC 3′, and antisense primer, 5′ TCC-AGG-AGC-TGA-TAA-GCA-TAG-AGA-GC 3′, and PCR was performed according to the Platinum Supermix protocol (Invitrogen).

3- **Immunostaining**. was done to detect eGFP. Salivary sections were stained overnight with a rabbit polyclonal antibody to mouse GFP (1∶100dilution; Aabcam # ab290), one hour with Rhodamine red–conjugated rabbit secondary antibody (Jackson ImmunoResearch), and then stained with DAPI.

*4-* **Focus score.** As previously described [7], half of a submandibular gland per mouse was fixed in 10% formalin and embedded in paraffin. Sections were cut at 5–8 µm thick and subsequently stained with hematoxylin and eosin (H&E). A score of 1 is a foci (aggregate) of at least 50 inflammatory cells per 4 mm^2^ of salivary tissue.

*5-* **Immunohistochemistry**. *(IHC):* Salivary sections were blocked for endogenous peroxidase (Dako # S2003) and incubated with normal rabbit serum (Dako # X0902). They were then stained with primary antibodies (CD4 # 550278, CD8 # 550281, CD19 # 550284, and CD49 # 559987, BD Pharmingen), CD11c (Abcam # ab33483), BAFF (Enzo-lifescience # ALX-804-131-400), and FoxP3 (eBioscience #14-5773-80, Clone FJK-16s) for 2 hours at room temperature. Polyclonal rabbit anti-Rat antibody biotinynlated, as secondary antibody, was applied for 1 hour. Visualization was done using the DAB system (Dako # K3468). Finally, sections were counterstained with hematoxylin (Fisher # BP2424-25 and methyl green (Dako # 1962). *Quantification of IHC result*: The surface area occupied by target cells was assessed under ×400 magnification by light microscopy for 3 tissue sections per gland. All focus scores per section were analyzed by NIH J Image software (NIH).

*6-* **Quantitative Real-Time PCR (qRT-PCR).** Gene expression analyses were performed using an Applied BioSystems quantitative Real Time PCR (qRT-PCR) system (model 7500). Total RNA was extracted from the submandibular glands with TRIZOL reagent (Invitrogen, Carlsbad, CA). The first-strand cDNA synthesis was performed by using Thermoscript RT-PCR system (Invitrogen, cat no # 11146-016) and qRT-PCR was done using 1 µg RNA per sample and TaqMan Universal Master Mix (Applied Biosystems). The probes and primers sequences were: Tumor necrosis factor alpha (*TNF-α*; Assay ID: Mm00443258), Epidermal Growth Factor (EGF; assay ID: Mm 00438696), Transforming Growth Factor-β (TGF-β; assay ID: Mm01178820), Aquaporin 5 (AQP5; Assay ID: Mm 437578), insulin like growth factor receptor 1 (IGF-IR; Assay ID: Mm00802841), (FGF-2; Assay ID: Mm00433287) and glyceraldehyde-3-phosphate dehydrogenase (*GAPDH* Assay ID: Mm99999915-g1), used as an endogenous reference running at 50°C for 2 min, 95°C for 10 min, and 40 cycles [95°C for 15 s, 60°C for 1 min]).

*7-* **Serum Preparation.** blood was drawn by cardiac puncture and held in a vertical tube for 15 minutes until it clotted. It was then centrifuged at 3000 RPM for 10 minutes and the serum removed.

### Statistical Analysis

To determine statistical significant differences (P<0.05), Linear Mixed Models and ANOVA analysis (Tukey's Post-Hoc test) were used. Subjects (data from mice) between and within the groups were compared at different time points using SPSS version 17 (IBM, USA).

## Supporting Information

Figure S1 **Saliva composition.** Total protein concentrations (A), EGF (B), and amylase activity (C) were not significantly different among the groups at week 8 (baseline) versus week 22 (end of experiment) (*P>*0.05). (n = 5 to 9 mice per group)
(TIF)

Figure S2 **Kaplan–Meier plot for normoglycemia.** Blood sugar levels in control (square), CFA (triangle), MSC (diamond) and MSC+CFA (circle) treated NOD mice were monitored for 22 weeks. All mice were normoglycemic from the start of the experiment (8 weeks) until 12 weeks of age. The first diabetic mouse was diagnosed in the control group (square) at week 13, and 60% of control mice developed diabetes at week 21 of age (* P<0.05). However, ninety percent of mice in MSC and MSC+CFA groups (circle and diamond) and eighty percent of mice in the CFA group were normoglycemic during the course of the experiment.
(TIF)

Figure S3 **Flow cytometry analysis of freshly isolated CD45^−^/Ter119^−^ cells (before being placed in culture).** Cells were stained for the following surface markers: CD45, Ter119, CD11B, Sca-1, CD106, CD105, CD73, CD29 and CD44. Data are representative of at least three separate experiments. This experiment shows 78.9% CD45^−^, 98% TER119^−^, 97.1% CD11b^−^ and 49.5% Sca1^+^, 60.8% CD106^+^, 57.6% CD105^+^, 47.3% CD73^+^, 89.7% CD29^+^, 37.7% CD44^+^.
(TIF)
