# Supplementary figures and images for: Mesenchymal Stromal Cells Improve Salivary Function and Reduce Lymphocytic Infiltrates in Mice with Sjögren's-Like Disease
Source: PLoS One. 2012 Jun 7;7(6):e38615. doi: 10.1371/journal.pone.0038615 (PMC3369846; doi:10.1371/journal.pone.0038615)

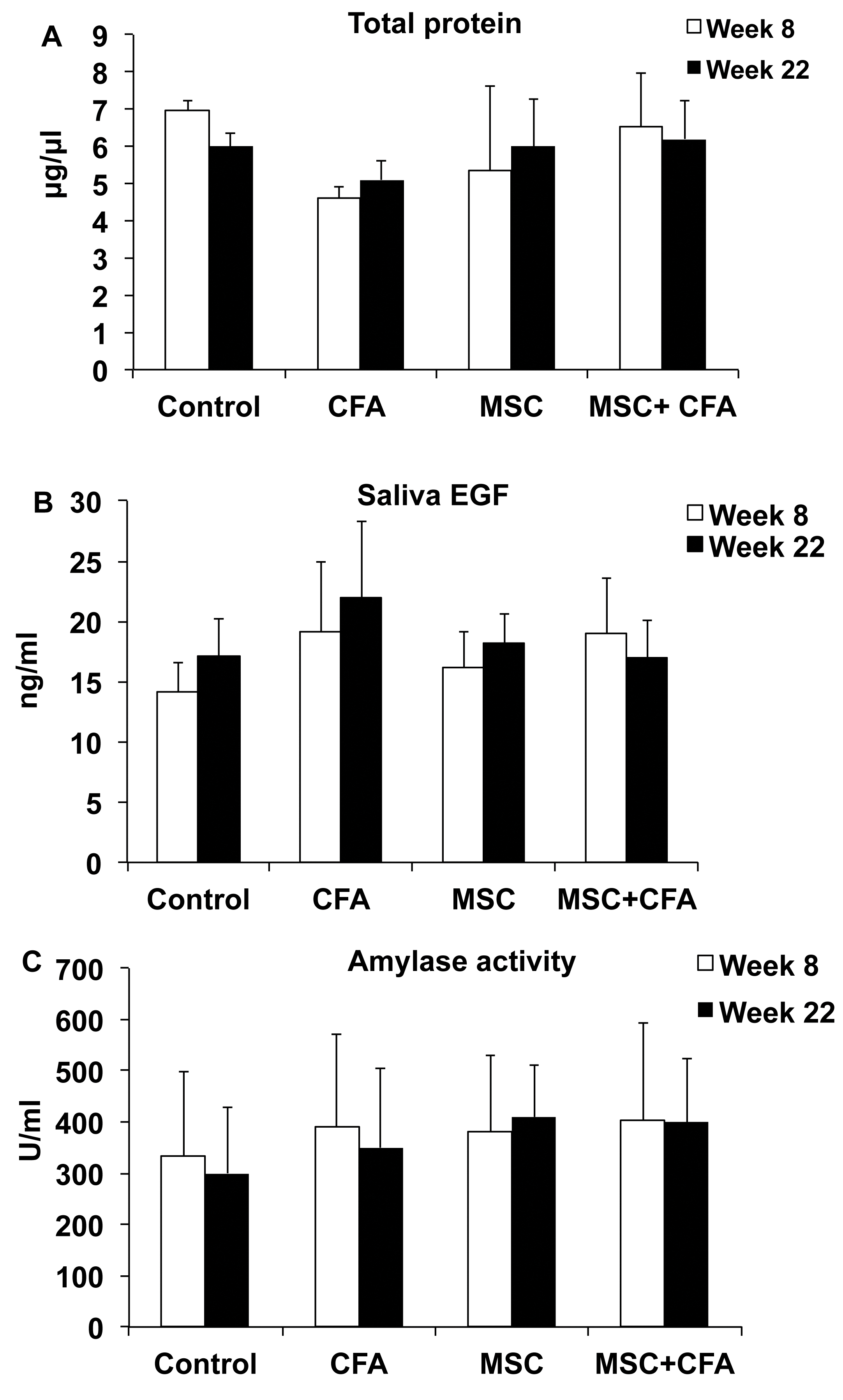

Supplement: Figure S1 — Saliva composition. Total protein concentrations (A), EGF (B), and amylase activity (C) were not significantly different among the groups at week 8 (baseline) versus week 22 (end of experiment) (P>0.05). (n = 5 to 9 mice per group) (TIF) [file pone.0038615.s001.tif]

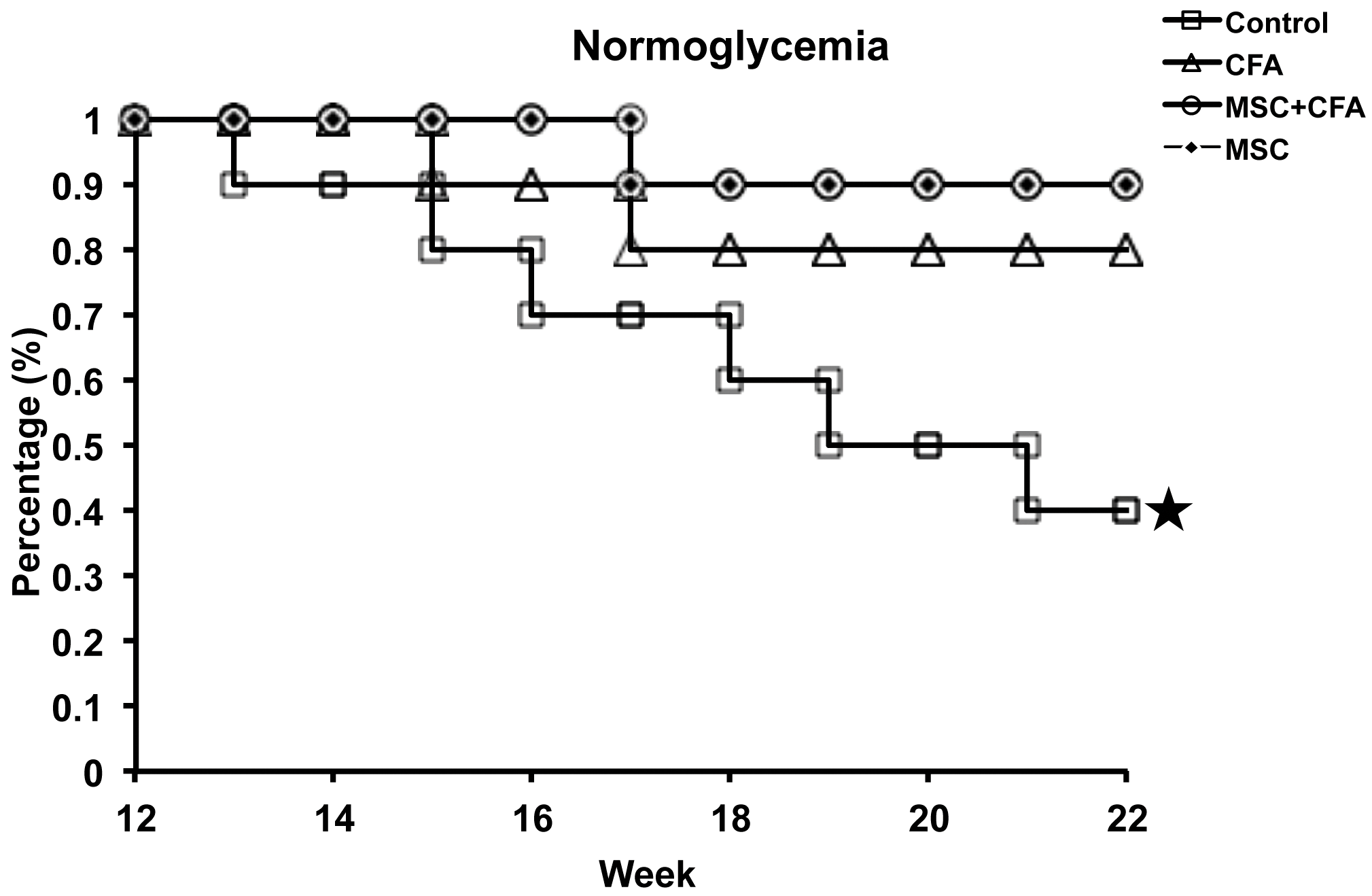

Supplement: Figure S2 — Kaplan–Meier plot for normoglycemia. Blood sugar levels in control (square), CFA (triangle), MSC (diamond) and MSC+CFA (circle) treated NOD mice were monitored for 22 weeks. All mice were normoglycemic from the start of the experiment (8 weeks) until 12 weeks of age. The first diabetic mouse was diagnosed in the control group (square) at week 13, and 60% of control mice developed diabetes at week 21 of age (* P<0.05). However, ninety percent of mice in MSC and MSC+CFA groups (circle and diamond) and eighty percent of mice in the CFA group were normoglycemic during the course of the experiment. (TIF) [file pone.0038615.s002.tif]

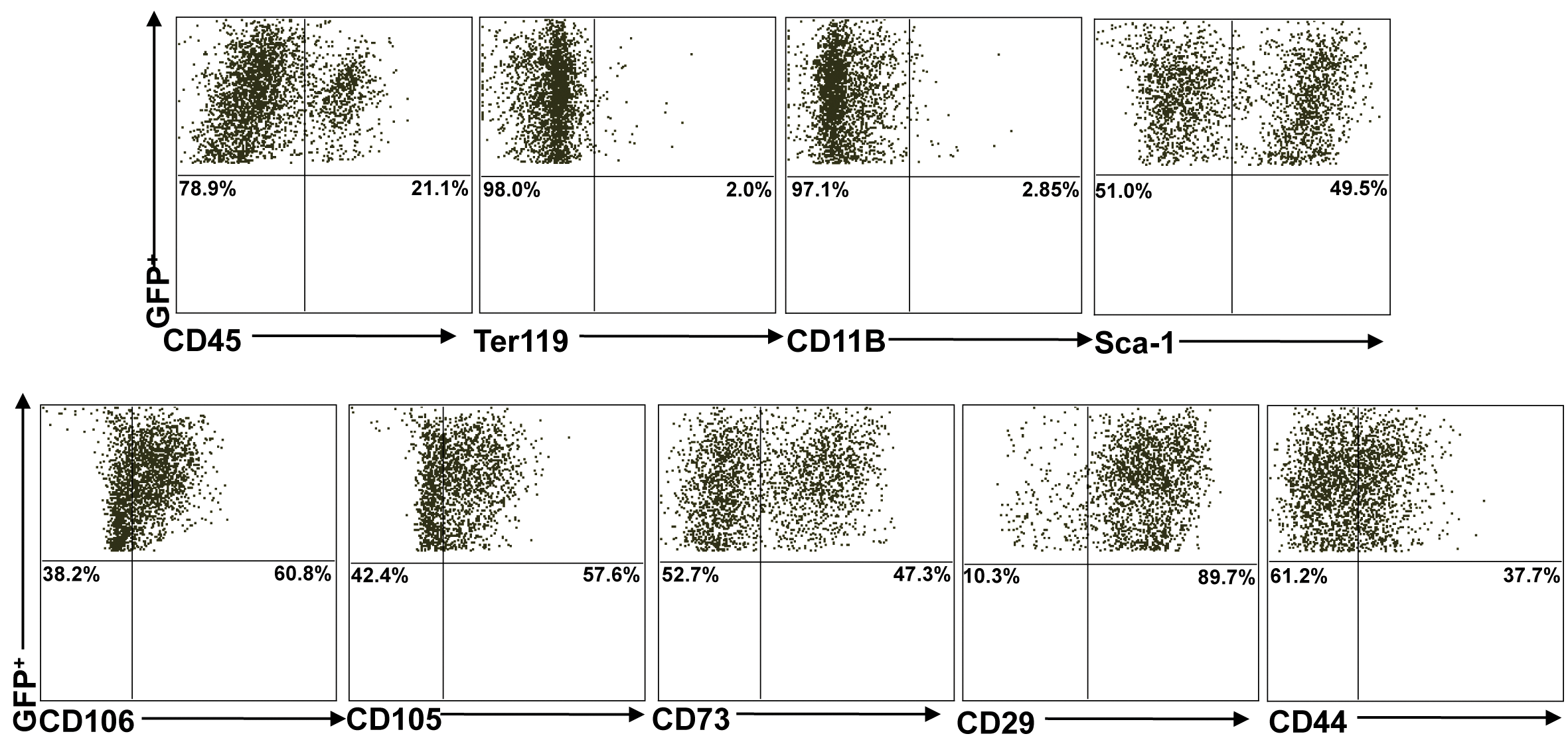

Supplement: Figure S3 — Flow cytometry analysis of freshly isolated CD45−/Ter119− cells (before being placed in culture). Cells were stained for the following surface markers: CD45, Ter119, CD11B, Sca-1, CD106, CD105, CD73, CD29 and CD44. Data are representative of at least three separate experiments. This experiment shows 78.9% CD45−, 98% TER119−, 97.1% CD11b− and 49.5% Sca1+, 60.8% CD106+, 57.6% CD105+, 47.3% CD73+, 89.7% CD29+, 37.7% CD44+. (TIF) [file pone.0038615.s003.tif]
